# Interfacial dielectric layer as an origin of polarization fatigue in ferroelectric capacitors

**DOI:** 10.1038/s41598-020-64451-0

**Published:** 2020-04-30

**Authors:** M. T. Do, N. Gauquelin, M. D. Nguyen, J. Wang, J. Verbeeck, F. Blom, G. Koster, E. P. Houwman, G. Rijnders

**Affiliations:** 1 MESA+ Institute for Nanotechnology, University of Twente, P.O. Box 217, Enschede, 7500 AE The Netherlands; 2 Electron Microscopy for Materials Science, University of Antwerp, 2020 Antwerp, Belgium; 3 Canon Production Printing Netherlands, P.O. Box 101, 5900 MA Venlo, The Netherlands; 4 Hanoi National University of Education, 136 Xuan Thuy, Cau Giay, Hanoi, Vietnam

**Keywords:** Materials for devices, Structural materials

## Abstract

Origins of polarization fatigue in ferroelectric capacitors under electric field cycling still remain unclear. Here, we experimentally identify origins of polarization fatigue in ferroelectric PbZr_0.52_Ti_0.48_O_3_ (PZT) thin-film capacitors by investigating their fatigue behaviours and interface structures. The PZT layers are epitaxially grown on SrRuO_3_-buffered SrTiO_3_ substrates by a pulsed laser deposition (PLD), and the capacitor top-electrodes are various, including SrRuO_3_ (SRO) made by *in-situ* PLD, Pt by *in-situ* PLD (Pt-inPLD) and *ex-situ* sputtering (Pt-sputtered). We found that fatigue behaviour of the capacitor is directly related to the top-electrode/PZT interface structure. The Pt-sputtered/PZT/SRO capacitor has a thin defective layer at the top interface and shows early fatigue while the Pt-inPLD/PZT/SRO and SRO/PZT/SRO capacitor have clean top-interfaces and show much more fatigue resistance. The defective dielectric layer at the Pt-sputtered/PZT interface mainly contains carbon contaminants, which form during the capacitor *ex-situ* fabrication. Removal of this dielectric layer significantly delays the fatigue onset. Our results clearly indicate that dielectric layer at ferroelectric capacitor interfaces is the main origin of polarization fatigue, as previously proposed in the charge injection model.

## Introduction

Ferroelectrics with two distinguishable polarized electrical states are widely used in various applications such as non-volatile ferroelectric random access memories^1,2^, micro-electro-mechanical systems^3^, and other advanced electronic devices^4,5^. These devices normally operate under a drive AC voltage, which means that integrated functional ferroelectric layers must withstand prolonged electric field cycling. This operation mode often leads to the so-called (ferroelectric) polarization fatigue, i.e. the loss of polarization of ferroelectric layer upon repeated electric field switching, which directly limits the stability of the host device. It is therefore important to gain further insight into origins of polarization fatigue, which may provide solutions to remedy the effect.

Numerous studies have explained characteristic behaviours of polarization fatigue and discussed its origins^6,7^. The most common behaviour of polarization fatigue in ferroelectric capacitor is its dependence on electrode material. Capacitors with conductive oxide electrodes such as SrRuO_3_^8,9^, LaNiO_3_^10,11^, LaSrMnO_3_^12^, IrO_2_^13^, LaSrCaO_3_^14^, YBa_2_Cu_3_O_x_^15,16^, and RuO_2_^17,18^ are generally free of fatigue for more than 10^12^ electric field cycles. In contrast, capacitors with conventional metal electrodes such as Pt and Au are strongly fatigued after only 10^3^ field cycles [ref. ^6^ and references therein]. One widely discussed model on fatigue origins is the so-called oxygen-vacancy hypothesis, which suggests that oxygen vacancies inside ferroelectric layers are the primary origin of polarization fatigue^19,20^. The oxygen vacancies were claimed to migrate and accumulate at the capacitor interfaces during field cycling, subsequently cause domain-wall pinning^21^ and polarization screening^22,23^, which lead to polarization fatigue. Metal electrodes were thought to block the migration of oxygen vacancies, resulting in a significant accumulation of these defects at the capacitor interfaces and leading to a pronounced fatigue. In contrast, oxide electrodes are believed to be transparent to the migration of oxygen vacancies, the fatigue effect in this kind of capacitor is therefore very small. Although the oxygen-vacancy hypothesis can qualitatively explain the dependence of polarization fatigue on electrode material, it cannot explain other reported behaviours of fatigue^6^. For instance, unipolar field cycling, which can also cause oxygen vacancy migration and subsequent accumulation at interfaces, is expected to significantly fatigue the capacitors; however, experimental data show that the fatigue under unipolar field cycling is much less than that under bipolar field cycling^24,25^. Moreover, a correlation between defect accumulation at capacitor interfaces and fatigue behaviour of the device under field cycling are still questionable^26,27^. In conclusion, native defects inside the ferroelectric layer such as oxygen vacancies only play a minor role in polarization fatigue.

Another popular argument on polarization fatigue is that the effect is indeed an interface-related phenomenon. Lou *et al*.^28^ assumed that a thin layer with a low dielectric constant (as compared to ferroelectric materials) is present at the metal electrode/ferroelectric interface. This interfacial layer is expected to induce a local depolarization field produced by unscreened, positive bound charges at the top of nucleating reverse domains arising from head-to-head polarization vectors. The depolarization field can therefore be at an order of MV’s/cm, which can cause a significant electron injection from the metal electrode into the ferroelectric layer at each polarization switching during field cycling. The associated electron injection can thermally decompose the ferroelectric layer, leading to suppression of polarization switching^29^. The interfacial dielectric layer was suggested not to present at oxide-electrode/ferroelectric interfaces so that such a capacitor is free of fatigue. Thus, the origin of polarization fatigue was proposed to be the dielectric layer at the electrode/ferroelectric interface, and the mechanism to be the domain switching inhibition caused by electron injection and ferroelectric degradation. The charge injection model is supported by some experimental data^30,31^. However, further direct evidences are needed to confirm the model. Detailed experimental investigations into the relation between fatigue behaviour and interface structure of ferroelectric capacitors would provide insights of fatigue origins. To the best of our knowledge no such investigation has been reported. Note that these experimental objectives are particularly challenging for sol-gel fabricated structures, which contain voids, grain boundaries, and undefined interfaces. Such study is more straightforward in epitaxial thin-film capacitors with dense microstructures and atomically sharp interfaces.

In this work, we aim to experimentally identify the major origin of polarization fatigue in ferroelectric thin-film capacitors. To realize this objective, we investigated fatigue behaviour and interface structure of PZT capacitors with different top electrodes, including SRO by PLD, Pt by *in-situ* PLD (Pt-inPLD), *ex-situ* PLD (Pt-exPLD), and *ex-situ* sputtering (Pt-sputtered). The PZT layers are epitaxially grown on SrRuO_3_-buffered SrTiO_3_ substrates, which allows us to characterize the capacitor interfaces at an atomic scale. We found that fatigue behaviour of the capacitor is directly related to the structure of the top-electrode/PZT interface. The Pt-sputtered/PZT/SRO and Pt-exPLD/PZT/SRO capacitor have a defective dielectric layer at their top interfaces, and these devices rapidly fatigued under field cycling. The Pt-inPLD/PZT/SRO capacitor has a clean top interface without any defective layer, and this capacitor fatigued much more later, with 10^4^–10^5^ times more field cycles. The SRO/PZT/SRO capacitor has atomically sharp epitaxial interfaces, which make the capacitor free of fatigue. The defective dielectric layer at the Pt-sputtered/PZT (and Pt-exPLD/PZT) interface contains carbon contaminants, which arise from the exposure of the PZT surface to ambient atmosphere during the *ex-situ* fabrications. *In-situ* removals of this defective layer significantly enhance fatigue resistance of the capacitor. Our experimental results clearly indicate that the presence of an interfacial dielectric layer is the major origin of polarization fatigue in ferroelectric capacitors, which was theoretically proposed in the charge injection model.

## Results

**Figure 1:**
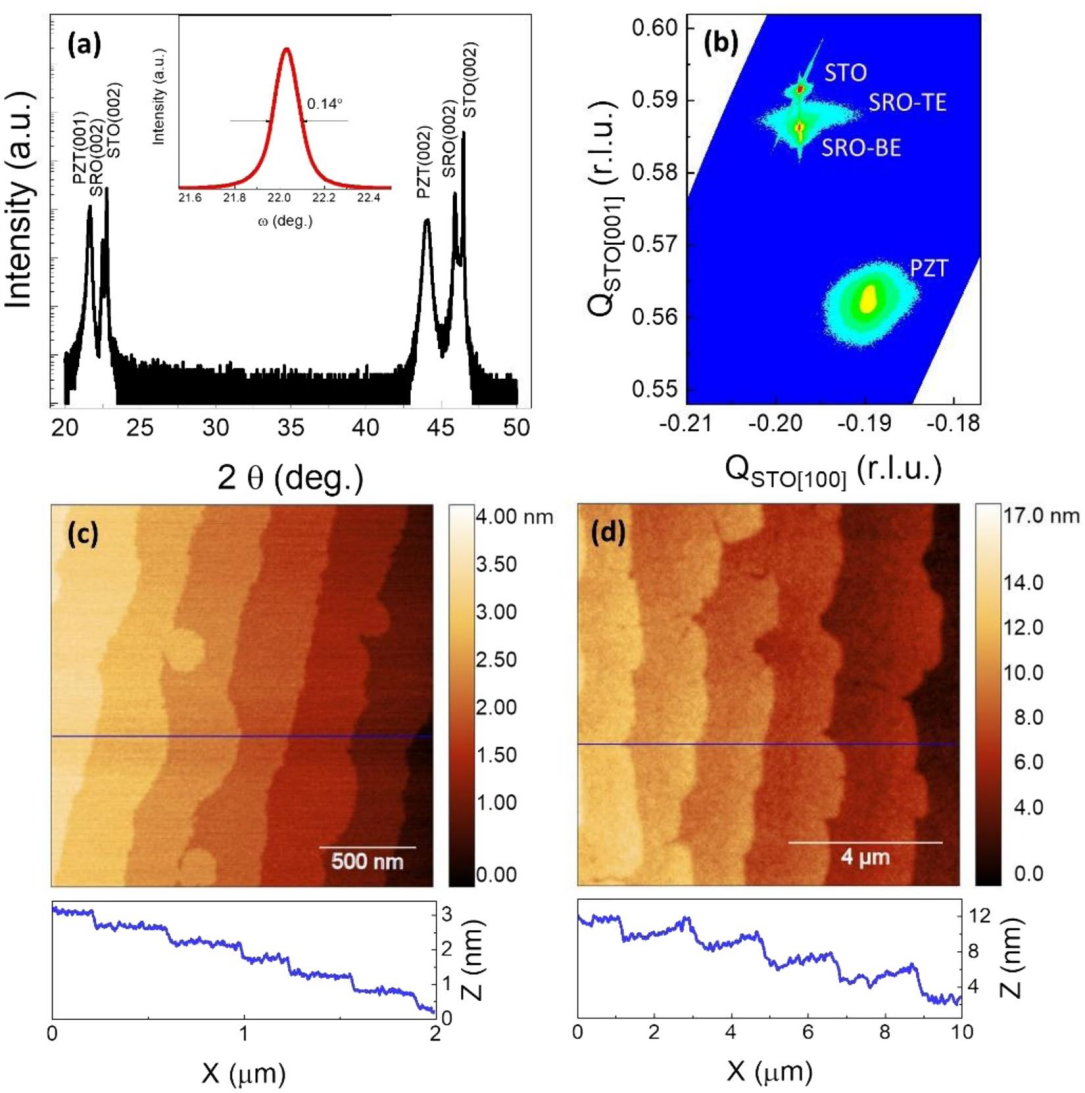
(**a**) XRD θ-2θ scan and rocking curve of PZT (002) reflection (inset). (**b**) Reciprocal space map around the PZT ($\bar{1}03$) reflection (TE and BE represent for top and bottom electrode, respectively). (**c,d**) AFM images of the SRO bottom electrode and the PZT layer, respectively.

Structure crystallization and surface morphology of the grown layers were characterized by X-ray diffraction (XRD) and atomic force microscopy (AFM), respectively (Fig. 1). The XRD θ–2θ scan shows that the PZT layer and SRO bottom electrode have been grown with a (001) out-of-plane orientation, following the orientation of the SrTiO_3_ (STO) substrate. The rocking curve of the PZT (002) reflection has a small FWHM (0.14°), reflecting a high crystalline quality of the PZT layer. The reciprocal space map (RSM) around the PZT ($\bar{1}$03) reflection indicates an epitaxial growth with a PZT[100] ǁ SRO[100] ǁ STO[100] in-plane relationship. AFM images show that the SRO bottom electrode is atomically flat with unit-cell stepped terraces of the STO substrate surface, indicating a step-flow growth of the SRO layer. Step bunching appears on the PZT surface, indicating a layer-by-layer growth of the PZT layer.

**Figure 2:**
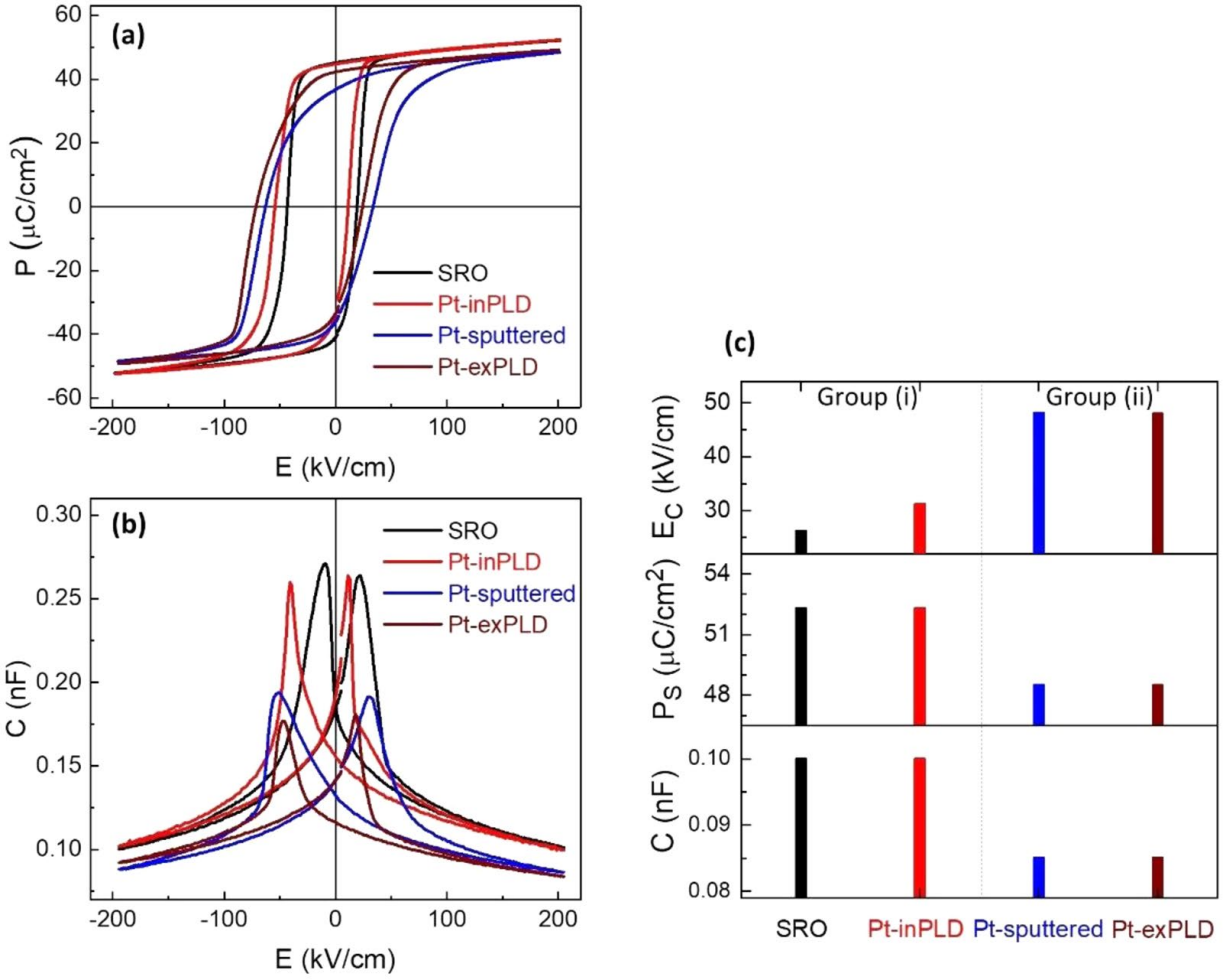
(**a**) P-E loops and **(b**) C-E curves of the PZT capacitors with different top electrodes. (**c**) Average coercive fields (E_C_), maximum polarizations (P_S_), and capacitances (C) of the capacitors.

Ferroelectric responses of the PZT capacitors with different top electrodes (SRO, Pt-inPLD, Pt-sputtered, and Pt-exPLD – see Methods section for capacitor fabrication details) were characterized by their P-E loops and capacitance-field (C-E) curves (Fig. 2). In general, all capacitors show square-shaped P-E loops, indicating well-developed ferroelectric behaviours. On the basis of the shape of the loops, one can divide the capacitors into two groups: (i) capacitors with SRO (S1) and Pt-inPLD (S2) top electrode, (ii) capacitors with Pt-sputtered (S3) and Pt-exPLD (S4) top electrode. There are three main differences in the P-E loop between these two groups. First, the capacitors in group (i) show a lower (average) coercive field ${E}_{C}=({E}_{C}^{+}+{E}_{C}^{-})/2$, (26.2 kV/cm for capacitor S1 and 31.2 kV/cm for capacitor S2) than those in the group (ii) (48.2 kV/cm for capacitor S3 and 48.0 kV/cm for capacitor S4). Second, the first group shows a maximum polarization P_S_ = 52.8 μC/cm^2^, slightly higher than that of the second group P_S_ = 48.5 μC/cm^2^ (all measured at 200 kV/cm). Third, the P-E loops of the second group are more slanted and rounded than those of the first group. We suspect that these different features are consequences of a presence of a non-ferroelectric layer at the interface of capacitor S3 (Pt-sputtered) and S4 (Pt-exPLD), which is absent in capacitor S1 (SRO) and S2 (Pt-inPLD). We note that all the loops show asymmetry along the field axis, which can be attributed firstly to the flexoelectric effect in the PZT layer with a strain gradient (in all capacitors) and secondly to a difference in work functions of the electrodes (in asymmetric capacitors).

The C-E curves of the capacitors are also sensitive to the top electrode. For capacitor S1 (SRO) and S2 (Pt-inPLD), the static capacitances at +/−200 kV/cm (where contribution from polarization switching are negligible) are almost similar, and determined by the intrinsic capacitance of the PZT layer only, ${{C}}_{{PZT}}\approx 100\,{pF}$. For capacitor S3 (Pt-sputtered) and S4 (Pt-exPLD), the static capacitances are lower, ${{C}}_{{tot}}\approx 85\,{pF}$. The capacitances of capacitor S3 and S4 can be attributed to the serial connection of the capacitance *C*_*PZT*_ of the PZT layer and capacitance *C*_*int*_ of the non-ferroelectric interfacial layer, ${{C}}_{{int}}={{\varepsilon }}_{0}{{\varepsilon }}_{{int}}{A}/{{d}}_{{int}}$, with ${\varepsilon }_{\mathrm{int}}$ and *d*_*imt*_ are the relative dielectric constant and thickness of the interfacial layer, and *A* is the area of the capacitor (${10}^{-8}{{\rm{m}}}^{2}$). Hence, one can have

1 $$\begin{array}{c}\frac{1}{{{C}}_{{tot}}}=\frac{1}{{{C}}_{{PZT}}}+\frac{1}{{{C}}_{{int}}}=\frac{1}{{{C}}_{{PZT}}}+\frac{{{d}}_{{int}}}{{{\varepsilon }}_{{int}}{{\varepsilon }}_{0}{A}}\\ \frac{{{\varepsilon }}_{{int}}}{{{d}}_{{int}}}=\frac{1}{{{\varepsilon }}_{0}{A}}{\left(\frac{1}{{{C}}_{{tot}}}-\frac{1}{{{C}}_{{PZT}}}\right)}^{-1}\approx 6.4\,({n}{{m}}^{-1})\end{array}$$

The numerical result holds for the capacitor S3 and S4, which has the Pt-sputtered and Pt-exPLD top electrode, respectively. Thus, if one can observe the (non-ferroelectric) interfacial layer in these capacitors, one can estimate dielectric constant of this layer from Eq. (1).

**Figure 3:**
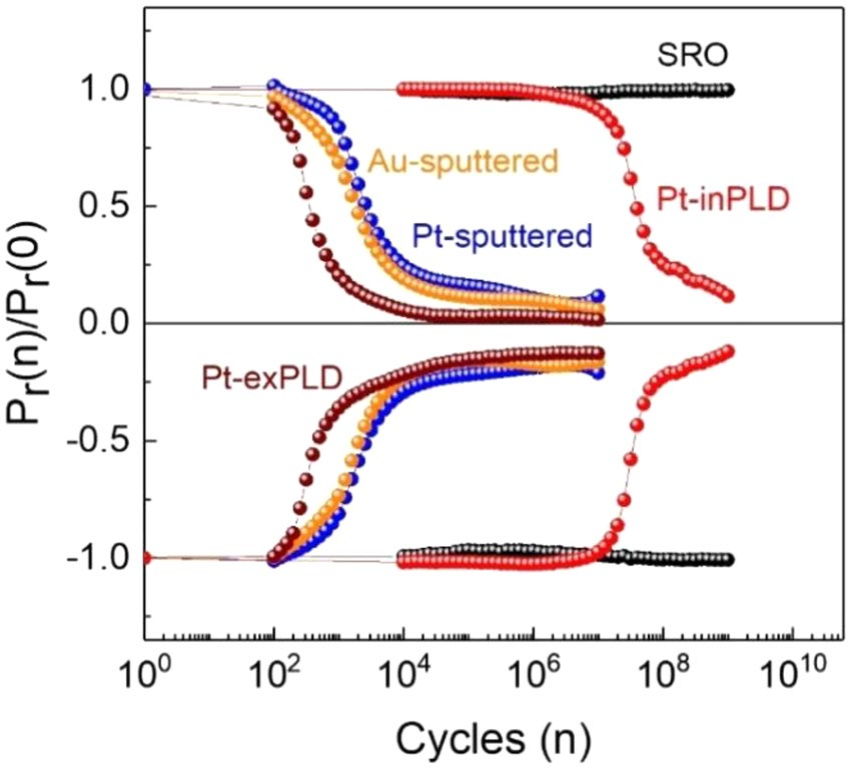
Fatigue behaviours of the PZT capacitors with different top electrodes under bipolar field cycling. The field cycling is at 200 kV/cm and 1 kHz.

Fatigue behaviour of the PZT capacitors under field cycling (with field amplitude of 200 kV/cm and cycling frequency of 1 kHz) is strongly dependent on the top electrode (Fig. 3). Capacitor S1 with both SRO electrodes is free of fatigue for over at least 10^9^ field cycles (further tests show that this type of capacitor is still non-fatigued up to 10^12^ cycles). Noted that capacitors with SRO top electrode made by *in-situ* PLD and *ex-situ* PLD show exactly similar ferroelectric and fatigue behaviours. Capacitor S2 with Pt-inPLD top electrode is free of fatigue up to 10^7^ cycles, but beyond this number of cycles it slowly fatigues (about 80% polarization loss within about 10^8^ cycles). We could not make capacitors with *in-situ* sputtered Pt (or Au) electrodes, but we believe that these capacitors show a similar fatigue as capacitor S2. Capacitor S3 (Pt-sputtered), S4 (Pt-exPLD), and S5 (Au-sputtered) show similar rapid fatigues with more than 80% polarization loss in about 10^3^−10^4^ cycles. The free fatigue of capacitor S1 with both SRO electrodes and the strong fatigue of capacitor S3 (and S4, S5) with conventional sputtered Pt (or Au) electrodes are well consistent with a large body of data in literature^6^. The additional information provided in this study, which is to our knowledge not available in literature, is that capacitor S2 with an *in-situ* fabricated metal electrode can be free of fatigue for 10^4^–10^5^ times more cycles than those with an *ex-situ* fabricated metal electrode.

**Figure 4:**
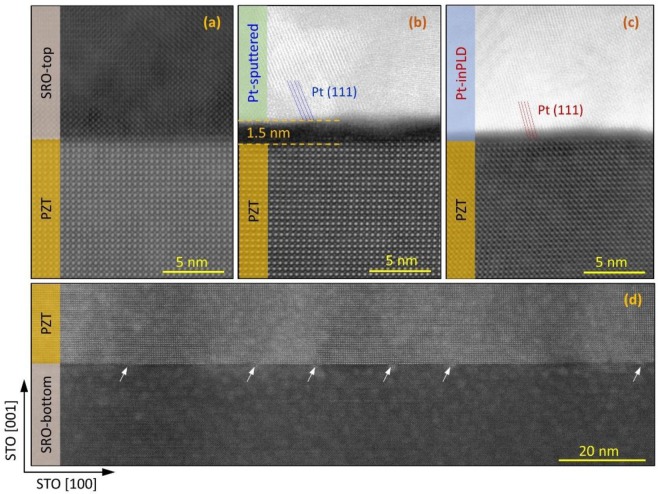
TEM investigations on the interfaces of the capacitors with different top electrodes: (**a–c**) SRO/PZT, Pt-sputtered/PZT, Pt-inPLD/PZT top interface, and (**d**) PZT/SRO bottom interface. The Pt-sputtered/PZT interface has a defective layer, which is absent at the Pt-inPLD/PZT interface. The SRO/PZT and PZT/SRO interface are atomically sharp because of epitaxial growth. The arrows point to misfit dislocations.

To characterize the capacitor interface structures, we performed cross-sectional transmission electron microscopy (TEM) on the pristine (before field cycling) capacitor S1 (SRO/PZT/SRO), S2 (Pt-inPLD/PZT/SRO), and S3 (Pt-sputtered/PZT/SRO) (Fig. 4). The PZT and SRO layers are of high crystalline quality, and the SRO/PZT interfaces are atomically sharp due to the epitaxial growth of these materials. Both the Pt-sputtered and the Pt-inPLD layer show clear (111) lattice planes, in accordance with XRD diffraction of these layers (not shown here). Most importantly, a clear defective layer is observed at the Pt-sputtered/PZT interface and not at the Pt-inPLD/PZT interface. This layer has a poor crystalline structure with an average thickness of about 1.5 nm. In the Pt-sputtered/PZT sample, the Pt lattice planes start from the top surface of the defective layer, whereas in the Pt-inPLD/PZT sample, the Pt lattice planes start directly from the top PZT atomic layer. We did not characterize the Pt-exPLD/PZT and the Au-sputtered/PZT interface; however, since their fatigue behaviours are similar to that of the Pt-sputtered/PZT capacitor, we expect that these interfaces have a similar defective interfacial layer. For all samples, the PZT/SRO bottom interfaces are atomically sharp with presence of misfit dislocations at regular spacings (marked by arrows), caused the in-plane lattice mismatch between PZT and STO (note that the bottom SRO layer is fully strained to the STO substrate).

With *d*_*int*_ = 1.5 *nm* in Eq. (1), one finds ${{\rm{\varepsilon }}}_{{int}}\approx 9.6$. This relative dielectric constant value of the interfacial layer is much smaller than the estimated value (from *C*_*PZT*_) of PZT, *ε*_*PZT*_ ≈ 280.

**Figure 5:**
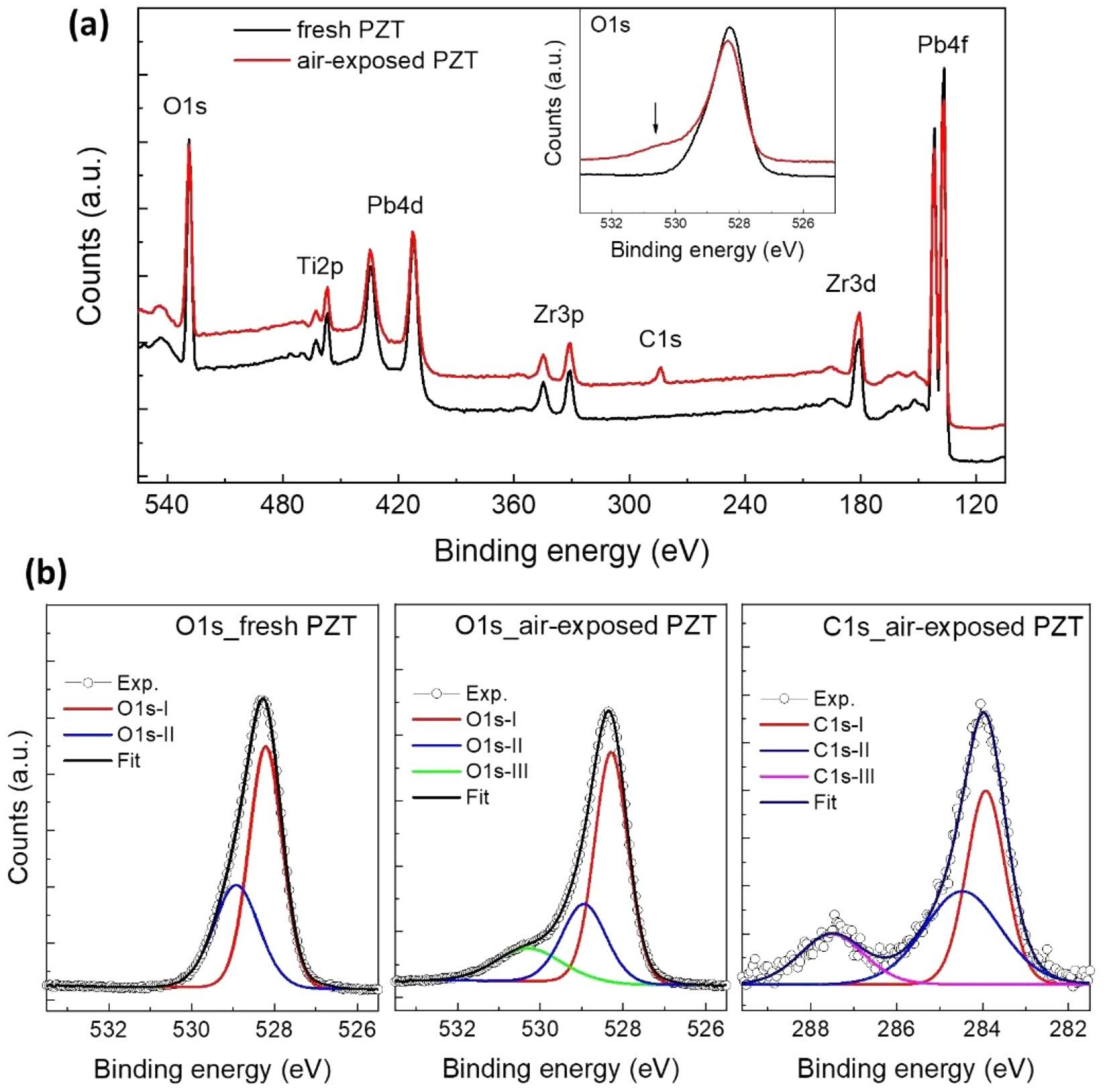
XPS on the fresh and air-exposed PZT surface. (**a)** Survey spectra with characteristic core levels (the inset shows high-resolution spectra of O 1 s). (**b**) High-resolution spectra of O 1 s and C 1 s with component peaks.

As shown above, the fatigue behaviour and the top interface structure of capacitor S2 (Pt-inPLD/PZT/SRO) and capacitor S3 (Pt-sputtered/PZT/SRO) are significantly different. These differences originate from the fabrication procedure of these capacitors. In the capacitor S3, the PZT surface has been exposed to ambient atmosphere before the Pt layer is deposited by a sputtering. In contrast, in the capacitor S2, the PZT surface remains in vacuum before the Pt layer is deposited by an *in-situ* PLD. We conducted X-ray photoelectron spectroscopy (XPS) measurements to characterize differences in the elemental compositions of a fresh PZT surface remaining in the vacuum (simulating the fabrication of capacitor S2) and an air-exposed PZT surface (simulating the fabrication of the capacitor S3) (Fig. 5). The survey spectra of the two PZT surfaces show clear characteristic peaks of Pb 4 f, Zr 3d, Ti 2p, and O 1 s core levels. Most importantly, we can observe a clear peak around 283.8 eV in the spectrum of the air-exposed PZT surface, and not in the spectrum of the fresh PZT surface. This peak corresponds to the C 1 s edge, indicating that, when the PZT layer has been exposed to air, carbon contaminants will be present on its surface. As seen from the high-resolution spectra, the air-exposed PZT surface shows an additional shoulder on the left side of the O 1 s main peak as compared to the one of the fresh PZT surface (Fig. 5(a) inset at arrow). For the other elements, there are no significant differences between the two samples. The O 1 s spectrum of the fresh PZT surface was decomposed into two component peaks at 528.3 eV (O 1s-I) and 528.9 eV (O 1s-II), which are attributed to lattice oxygens and surface adsorbed oxygens, respectively^32,33^. Apart from these two components, the O 1 s spectrum of the air-exposed PZT surface shows an additional left-side shoulder peak at 530.3 eV (O 1s-III), which is attributed to carbon surface contaminants^32^. The C 1 s spectrum was decomposed into three components at 283.8 (C 1s-I), 284.5 (C 1s-II), and 287.5 eV (C 1s-III), which are attributed to sp_3_ bulk C-C, C-O, and C=O bonds, respectively^33^. We therefore conclude that a carbon contamination layer is present at the Pt-sputtered/PZT interface in capacitor S3, which was previously shown in the TEM image as the non-crystalline defective layer. In contrast, there is no such contamination layer at the Pt-inPLD/PZT interface in the capacitor S2, which is also consistent with the TEM data. The defective layer at the Pt-sputtered/PZT interface consists of carbon contaminants, which have relative dielectric constant values of the order of 10 or less, which is consistent with the value ${{\varepsilon }}_{{int}}\approx 9.6$ estimated from the C-E measurements above.

**Figure 6:**
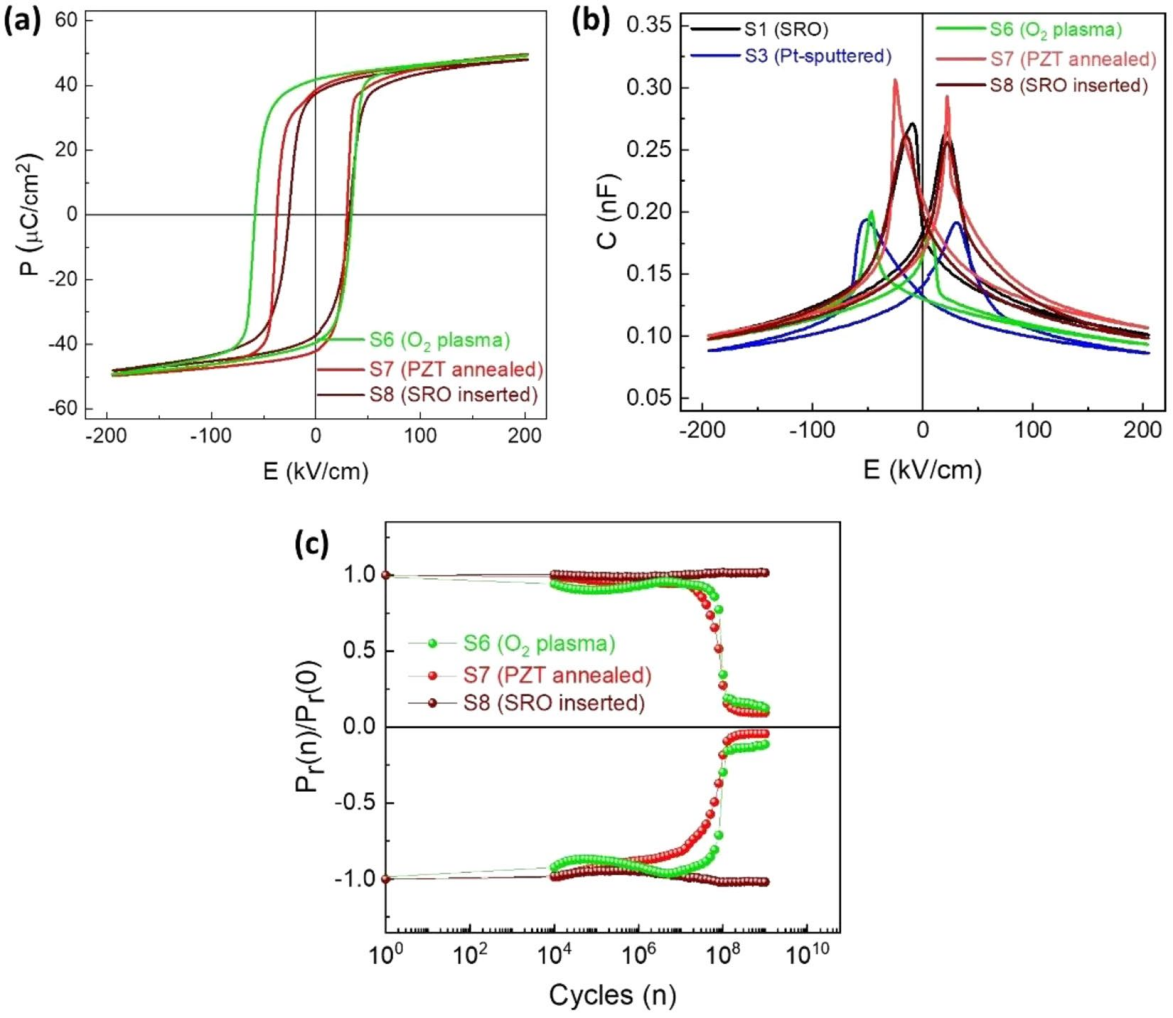
(**a**) P-E loops, (**b**) C-E curves, and **(c**) fatigue behaviours of the capacitors with the (air-exposed) PZT surface treated by O_2_ plasma and annealing, and a capacitor with a 5-nm SRO layer inserted at the Pt-inPLD/SRO interface.

The better fatigue resistance of capacitor S2 (Pt-inPLD/PZT/SRO) as compared to capacitor S3 (Pt-sputtered/PZT/SRO), S4 (Pt-exPLD/PZT/SRO), and S5 (Au-sputtered/PZT/SRO) indicates that a clean PZT top surface, which is in contact with the top electrode, is crucial to the fatigue behaviour of the capacitor. To further prove this argument, we tested fatigue behaviour of capacitors in which the air-exposed PZT surfaces were *in-situ* cleaned before deposition of metal top electrodes. In one sample, an *in-situ* O_2_ plasma was applied to the exposed PZT surface before sputtering an Pt top electrode, named as capacitor S6. In another sample, the air-exposed PZT/SRO stack was annealed at 400 °C for 30 minutes in O_2_ atmosphere in the PLD chamber before deposition of the Pt top electrode by PLD, named as capacitor S7. In the next sample, a 5-nm thick SRO layer was introduced between the Pt-inPLD electrode and the PZT layer, named as capacitor S8. Ferroelectric and fatigue behaviour of these capacitors are shown in Fig. 6. The capacitors at the pristine state show a good ferroelectric response, as indicated by their square-shaped P-E loops. Noted that the capacitor S6 (O_2_ plasma) shows a larger coercive field than the other capacitors, indicating that ferroelectric behaviour is sensitive to the PZT surface treatment. The capacitors with treated PZT surfaces show higher capacitances than capacitor S3 (Pt-sputtered/PZT/SRO) and they are closed to capacitor S1 (SRO/PZT/SRO). More importantly, the capacitor S6 and S7 with the (air-exposed) PZT surface treated before metal top electrode deposition show a fatigue resistance similarly to that of capacitor S2 (Pt-inPLD). No significant polarization loss is observed until 10^7^ cycles. These data indicate that the O_2_ plasma and oxygen annealing can remove the carbon contamination layer on the air-exposed PZT surface^34^, leading to a clean Pt/PZT interfaces. This also explains why both SRO top-electrodes by *in-situ* and *ex-situ* PLD, which were grown at 600 °C, provide exactly similar behaviours. The capacitor S8 with a 5-nm SRO buffer layer between the Pt-inPLD electrode and the PZT layer is free of fatigue, similarly to capacitor S1 with both SRO electrodes. This demonstrates again that the interface between the electrode and ferroelectric layer is crucial to fatigue resistance of the device.

## Discussion

We have shown that the top-electrode/PZT interface directly determines the fatigue behaviour of the PZT capacitor (Table 1). A defective, low dielectric constant layer at the Pt-sputtered/PZT (Au-sputtered/PZT and Pt-exPLD/PZT) interface makes a such capacitor strongly fatigued upon only 10^2^–10^3^ field cycles. The Pt-inPLD/PZT interface is clean without defective layer, consequently this capacitor is fatigue resistant until 10^7^ field cycles, which is a factor 10^4^–10^5^ times longer than the capacitors with *ex-situ* metal electrodes. In the ideal case of SRO/PZT/SRO capacitor, the atomically sharp SRO/PZT interfaces due to epitaxial growth make the capacitor perfectly free of fatigue for over 10^9^ field cycles. The strong dependence of fatigue behaviour of our PZT capacitors on the interface structure directly confirms that polarization fatigue is an interface-related effect^6^.

**Table 1:** Investigated PZT capacitors with various top electrodes and their fatigue behaviours.

Sample	Top electrode (TE)	Deposition technique	PZT surface treatment before TE deposition	Top interface structure	Fatigued after n cycles
S1	SRO	*In-situ* PLD	No	Epitaxial	>>10^9^
S2	Pt	*In-situ* PLD	No	Clean	10^7–8^
S3	Pt	*Ex-situ* sputter	No	Defective layer	10^2–3^
S4	Pt	*Ex-situ* PLD	No	Defective layer	10^2–3^
S5	Au	*Ex-situ* sputter	No	Defective layer	10^2–3^
S6	Pt	*Ex-situ* sputter	O_2_-plasma cleaning	Clean	10^7–8^
S7	Pt	*Ex-situ* PLD	Annealing	Clean	10^7-8^
S8	Pt	*In-situ* PLD	5 nm SRO by PLD	Epitaxial	>>10^9^

Our data experimentally support the charge injection model suggested by Lou *et.al*.^28^, which proposed that a thin dielectric layer presents at metal-electrode/ferroelectrics interfaces and that this interfacial layer is the primary cause of polarization fatigue of the capacitor. It is hard to observe such a thin layer in a frame of crystalline nano-grains and corrugated interfaces in sol-gel fabricated samples. Here, by working on the PZT epitaxially grown layers, we directly observe a defective layer of 1.5 nm at the top interface of the strongly fatigued Pt-sputtered/PZT/SRO capacitor. In the fatigue-resistant capacitors, such as SRO/PZT/SRO and Pt-inPLD/PZT/SRO, there is no a such defective layer at their interfaces. The defective interfacial layer consists of carbon contaminants, which have an average dielectric constant *ε*_*i*_ ≈ 9.6, much lower than that of bulk PZT (*ε*_*PZT*_ ≈ 280). At the Pt-sputtered/PZT interface, a net local transient depolarization field *E*_*bc*_ produced by the positive bound charges (${\delta }={{P}}_{{PZT}}\approx 0.4\,{C}/{{m}}^{2}$) in the reverse domains can be estimated through Lou’s model^28^.

$${E}_{bc/i}\approx \frac{{\sigma }}{{{\varepsilon }}_{i}.{{\varepsilon }}_{0}}\approx \frac{{P}_{PZT}}{{{\varepsilon }}_{{\rm{i}}}.{{\varepsilon }}_{0}}$$

With the presence of a dielectric layer with *ε*_*i*_ ≈ 9.6, one gets an extremely large depolarization field at the Pt-sputtered/PZT interface, ${E}_{bc/i}\approx 50\,MV/cm$,. At the SRO/PZT interface without a dielectric layer, with *ε*_*PZT*_ ≈ 280, the depolarization field then ${E}_{bc/0}\approx 1.2\,MV/cm$, much lower than the former case. The depolarization field can accelerate injection of electrons at the interface following Fowler-Nordheim tunneling mechanism

$$J=\frac{{e}^{3}{E}_{bc}^{2}}{8\pi h{\varphi }_{B}}exp\left(-\frac{8\pi {(2{m}^{\ast })}^{1/2}}{3eh}\frac{{(e{\varphi }_{B})}^{3/2}}{{E}_{bc}}\right)$$

*φ*_*B*_ and ${{\rm{m}}}^{\ast }$ are the barrier height and electron effective mass at the interface, respectively. Assuming that these parameters are the same for the Pt-sputtered/PZT and SRO/PZT interface, one obtains

$${J}_{i}/{J}_{0} \sim {({\varepsilon }_{PZT}/{\varepsilon }_{i})}^{2}exp(A({\varepsilon }_{PZT}-{\varepsilon }_{i}))$$

With

$$A=\frac{8\pi {(2{m}^{\ast })}^{1/2}}{3eh}\frac{{(e{\varphi }_{B})}^{3/2}{\varepsilon }_{0}}{P}$$

Consider that $e{\varphi }_{B}=1\,eV$ and ${m}^{\ast }={m}_{e}$, the free electron mass, then we obtain ${J}_{i}/{J}_{0}\approx {10}^{20}$. Thus, electron injection at the defective Pt-sputtered/PZT interface is significantly larger than that at the epitaxial SRO/PZT interface.

How do the injected electrons can be involved into polarization fatigue of the Pt-sputtered/PZT/SRO capacitor? Lou *et al*.^28^ proposed that the injected electrons can thermally decompose the ferroelectric structure nearby the interfaces, leading to inhibition of domain nucleation and consequently to the polarization loss. This mechanism likely happens in sol-gel PZT capacitors as the structure is delaminated during field cycling^29^. However, we did not observe any delamination in our cycled PZT capacitors. A possible reason is that the epitaxial PZT layer with a dense microstructure would has a larger heat conduction than a sol-gel PZT layer, which generally contains many voids and lattice defects. As a result, local heat accumulation in our capacitor interfaces due to the injected electrons is expected to be significantly less than in sol-gel films^29^, ceramic structures^35,36^, and organic layers^37^. Although our data support the model of charge injection at the metal/ferroelectric interface, we currently cannot draw a definitive conclusion on the subsequent mechanism of the fatigue. Together with structure degradation induced by the charge injection (if it happens), we suspect that injected electrons may be trapped at the defective Pt-sputtered/PZT interface, forming strong polarization pinning centers, which can inhibit the polarization domain switching of the PZT layer^38–40^. Further investigations into characteristic behaviours of fatigue such as its dependence on field cycling conditions, as well as into the interface structure of the capacitor at different fatigued states, need to be performed^41^.

As shown above, *in-situ* fabrications (by PLD or sputtering) of metal electrodes and *in-situ* cleanings (by O_2_ plasma or annealing) of ferroelectric layer surface improve the quality of the capacitor interfaces, which consequently enhance fatigue resistance of the capacitor. However, such a capacitor is still less fatigue resistant than the capacitor with both SRO electrodes. This can be understood by some possible reasons. First, the deposition of metal electrodes by either sputtering or PLD may cause some modifications on the top atomic layers of the ferroelectric film. By *in-situ* XPS measurements, McIntyre and co-workers^42,43^ reported on the formation of metallic Pb on PbTiO_3_ surfaces during the deposition of sputtered Pt. Similar observations were also reported by Chen *et al*.^44,45^. It was claimed that the heat of condensation of Pt atoms on the PZT surface decomposes Pb^2+^ into Pb^0^ metal. It is therefore that the top ferroelectric surface which is in contact with the metal electrode always contains lattice defects and elemental vacancies, which can subsequently contribute to polarization fatigue of the capacitor. Second, we conjecture that the type of bonding between the ferroelectric layer and the electrode plays a crucial role in fatigue resistance of the capacitor. A strong chemical bonding not only enhances the adhesion between the layers, but also reduces the number of lattice defects at the interface which lately might be involved into fatigue mechanisms. Indeed, a strong chemical bonding is formed in the continuous oxygen lattice across oxide-electrode/ferroelectric interfaces, which is contrast with a week bonding at metal/ferroelectric interfaces. A direct chemical bonding between Pt (Au) electrodes and perovskite ferroelectrics is not possible, but the use of an adhesion layer such as *in-situ* deposited Ti layer is expected to bring a certain positive effect. Metal bonding of the Pt (Au) electrode with the Ti layer and Ti-O bonding of the Ti layer with the ferroelectric layer can be formed, expecting to reduce the fatigue of the capacitor.

Practically, this work provides a way to improve fatigue resistance of metal-electrode ferroelectric capacitors, by using *in-situ* fabricated metal electrodes (or *ex-situ* electrodes but accompanied with a prior surface cleaning of ferroelectric layers). Pt and Au are still the most common electrodes in technical devices because of their high conductivity and chemical inertness. Moreover, metal electrodes provide better mechanical contacts with metal wiring. Therefore, although the fatigue resistance of these devices (10^7^ cycles) is less than that of the devices with SRO (or other conductive oxides) electrodes (at least 10^9^ cycles), the results are very promising for applications.

## Conclusion

We have experimentally investigated fatigue behaviour and interface structure of PZT thin-film capacitors with various top electrodes. Our data showed that a defective dielectric layer at metal/ferroelectric interface is the major origin of polarization fatigue in ferroelectric capacitors. Fatigue resistance of ferroelectric capacitors will be enhanced if the electrode/ferroelectric interface is clean without the defective layer. These findings directly support the charge injection model, which suggests that electron injection is significantly induced at the defective interface, giving rise to fatigue of the capacitor. In practice, *in-situ* processing of metal electrodes and/or *in-situ* cleanings of ferroelectric-layer surface before electrode deposition can be employed to improve the interface purity, which subsequently enhance the fatigue resistance of metal-electrode ferroelectric devices.

## Methods

### Thin-film capacitor fabrications

Epitaxial layers were grown by a pulsed layer deposition (PLD) technique^46^. A 100-nm thick SrRuO_3_ (SRO) layer was first grown on a TiO_2_-single-terminated SrTiO_3_ (STO) (001)-oriented substrate^47^ as bottom electrode. Substrate temperature and deposition pressure of the SRO layer were 600 °C and 0.13 mbar oxygen, respectively. A 250-nm thick PbZr_0.52_Ti_0.48_O_3_ (PZT) layer was subsequently grown on top of the SRO layer at 600 °C and 0.10 mbar oxygen. The small lattice mismatch between SRO (3.93 Å) and PZT (4.10 Å) allows the PZT layer to grow epitaxially with only few interfacial dislocations. After deposition, the films were cooled down to room temperature in a 1 bar oxygen atmosphere with a ramp rate of 8 °C/min. For capacitor top electrodes, either SRO or Pt was used. In the first case, the SRO top layer was grown on top of the PZT layer by an *in-situ* PLD with the same conditions as the SRO bottom layer. The SRO electrodes were then defined by photolithography combined with Ar ion etching (home-built RIBE system, NanoLab, MESA+). In the latter case, the Pt layers were made by different methods, including *in-situ* PLD, *ex-situ* sputtering, and *ex-situ* PLD.

For the capacitor with Pt top electrodes made by *in-situ* PLD (Pt-inPLD), the PZT/SRO stack was cooled down in the PLD chamber to room temperature then a 100 nm thick Pt layer was deposited by PLD at a pressure of 0.1 mbar Ar. Subsequent photolithography and Ar ion etching were performed to define the Pt electrode areas.

Sputtered Pt electrode is commonly used in practical devices because of its simple and high throughput fabrication. It has been also used in previous studies on fatigue of ferroelectric capacitors [ref. ^6^ and references cited therein]. The PZT/SRO stack was transferred from the PLD chamber to the cleanroom where a standard photolithography was used to make a photoresist pattern on top of the PZT surface. A Pt layer of 100 nm was then deposited by room temperature DC-magnetron sputtering (home-built T’COathy sputter system, NanoLab, MESA+) on top of the structure. The Pt electrode areas were obtained by lifting off the photoresist. For comparison, a capacitor with sputtered Au top electrodes (Au-sputtered) was also made in the same way.

For the capacitor with Pt top electrodes made by *ex-situ* PLD (Pt-exPLD), the PZT/SRO stack was taken from the PLD chamber and remaining in ambient atmosphere for 2 hours. The stack was then replaced in the PLD chamber again and the Pt layer was deposited by PLD at room temperature and a pressure of 0.1 mbar Ar. Photolithography and Ar ion etching were used to define the Pt electrodes.

### Sample characterizations

Crystalline structure and surface morphology of the layers were checked by X-ray diffraction (XRD, Philips X’Pert X-ray diffractometer) and atomic force microscopy (AFM, Bruker Dimension Icon) using a standard tapping mode, respectively.

Ferroelectric properties were measured with an aixACCT TF-2000 Analyzer. Fatigue behaviour was investigated under bipolar rectangular electric field cycling with an amplitude of 200 kV/cm and a cycling frequency of 1 kHz. At fixed cycle number intervals, polarization hysteresis (P-E) loops of the capacitor were measured using a triangular electric field with 200 kV/cm amplitude and 1 kHz frequency. Capacitance-field (C-E) curves of the capacitors were measured with a slow scanning staircase DC bias combined with a low-field (2.6 kV/cm) AC modulation with a frequency of 10 kHz. In all electrical experiments, the capacitor bottom electrode was always grounded.

X-ray photoelectron spectroscopy (XPS) measurements were performed to characterize the surface of PZT layers. An Omicron XM-1000 monochromated Al-Kα source was used with a pass energy to the detector of 20 eV. The angle between the sample surface normal and the detector was 1°. The recorded core-level spectra of the elements were then analysed by decomposition into Gaussian peak components after subtraction of the background signal. Transmission electron microscopy (TEM) was performed to characterize interfaces between PZT layers and electrodes using the X-Ant-Em instrument at the University of Antwerp. Cross-sectional cuts on the samples along the [001] direction of the STO substrate were prepared by a FEI Helios 650 dual-beam focused ion beam device.
